# Class-switched B cells display response to therapeutic B-cell depletion in rheumatoid arthritis

**DOI:** 10.1186/ar2686

**Published:** 2009-05-06

**Authors:** Burkhard Möller, Daniel Aeberli, Stefan Eggli, Martin Fuhrer, Istvan Vajtai, Esther Vögelin, Hans-Rudolf Ziswiler, Clemens A Dahinden, Peter M Villiger

**Affiliations:** 1 Clinic for Rheumatology, Clinical Immunology, and Allergology, Inselspital – University Hospital of Bern, Freiburgstraße, Bern 3010, Switzerland; 2 Department of Orthopaedic Surgery, Inselspital – University Hospital of Bern, Freiburgstraße, Bern 3010, Switzerland; 3 Section of Neuropathology, Institute of Pathology, University of Bern, Murtenstraße 31, Bern 3010, Switzerland; 4 Department of Orthopaedic, Plastic and Reconstructive and Hand Surgery, Inselspital – University Hospital of Bern, Freiburgstraße, Bern 3010, Switzerland; 5 Institute of Immunology, Inselspital – University Hospital of Bern, Freiburgstraße, Bern 3010, Switzerland

## Abstract

**Introduction:**

Reconstitution of peripheral blood (PB) B cells after therapeutic depletion with the chimeric anti-CD20 antibody rituximab (RTX) mimics lymphatic ontogeny. In this situation, the repletion kinetics and migratory properties of distinct developmental B-cell stages and their correlation to disease activity might facilitate our understanding of innate and adaptive B-cell functions in rheumatoid arthritis (RA).

**Methods:**

Thirty-five 'RTX-naïve' RA patients with active arthritis were treated after failure of tumour necrosis factor blockade in an open-label study with two infusions of 1,000 mg RTX. Prednisone dose was tapered according to clinical improvement from a median of 10 mg at baseline to 5 mg at 9 and 12 months. Conventional disease-modifying antirheumatic drugs were kept stable. Subsets of CD19^+ ^B cells were assessed by flow cytometry according to their IgD and CD27 surface expression. Their absolute number and relative frequency in PB were followed every 3 months and were determined in parallel in synovial tissue (n = 3) or synovial fluid (n = 3) in the case of florid arthritis.

**Results:**

Six of 35 patients fulfilled the European League Against Rheumatism criteria for moderate clinical response, and 19 others for good clinical response. All PB B-cell fractions decreased significantly in number (*P *< 0.001) after the first infusion. Disease activity developed independently of the total B-cell number. B-cell repopulation was dominated in quantity by CD27^-^IgD^+ ^'naïve' B cells. The low number of CD27^+^IgD^- ^class-switched memory B cells (MemB) in the blood, together with sustained reduction of rheumatoid factor serum concentrations, correlated with good clinical response. Class-switched MemB were found accumulated in flaring joints.

**Conclusions:**

The present data support the hypothesis that control of adaptive immune processes involving germinal centre-derived, antigen, and T-cell-dependently matured B cells is essential for successful RTX treatment.

## Introduction

B-cell depletion with the chimeric anti-human CD20 IgG_1 _antibody rituximab (RTX) represents a novel target-specific treatment option [1-3] for active rheumatoid arthritis (RA). RTX leads to almost total depletion of peripheral blood (PB) B cells for several months [1-6]. The subsequent clinical course follows the autoantibody kinetics more closely than the B-cell numbers in the blood [7]. Despite its specific mode of action on B cells, clinical response to RTX is not restricted to rheumatoid factor (RF)-positive or otherwise autoantibody-positive RA patients [2]. Important innate immune functions of B cells such as antigen presentation and cytokine production [8,9], but also B-cell-dependent adaptive autoimmune processes that were not represented by standard autoantibodies [10], are alternative explanations for this phenomenon.

Up to five repetitive B-cell depletion courses appear safe in RA [11,12], but the risk of secondary immunodeficiency with more repetitive RTX courses is still not ruled out. This uncertainty may cause restriction in re-treatment scheduling and requires at least ongoing surveillance [12-15]. There is a large variability in duration of response after RTX administration. Fixed short re-treatment intervals neglect the potential of saving immunosuppression and costs provided by this variability, whereas long intervals imply the risk of avoidable relapses and disease progression. Previous experimental studies indicated a rationale for repetitive RTX scheduling based on B-cell kinetics [5,6,16], but variable time lag between B-cell repopulation and clinical flare limited the immediate clinical application of B-cell repletion monitoring. Individual re-treatment intervals, therefore, are still recommended on the basis of the clinical course [17].

Which B-cell subset should be monitored? Long-lived plasma cells currently are believed to play a pivotal role in chronic autoimmunity [18]. They derive from short-lived plasma cells and undergo apoptosis unless they find survival niches of limited number in the bone marrow. Their progenitors, the CD19^+ ^plasmablasts, have undergone class switch on their differentiation pathway to further develop to antibody-producing CD19^- ^plasma cells. Plasmablasts draw a dynamic picture of ongoing autoimmune response in animal models [19]. They share CD27 positivity and IgD negativity with germinal centre (GC)-derived, affinity matured, CD27^+^IgD^- ^immunoglobulin (Ig) class-switched memory B cells (MemB). However, splenic long-lived plasma cells may also derive from extrafollicular maturation [20]. As long-lived plasma cells are primarily resistant to RTX due to a lack of CD20 expression, they currently are hard to be directly extinguished by any available therapeutic modality [18]. Plasma cells, in principle, are able to persist in tertiary immune organs, as it may be under certain circumstances the inflamed synovium [9,18]. Their number indeed was reported to be unchanged in the synovium 4 weeks after RTX [21] but strongly reduced later on [22-24]. Plasma cell numbers are very low after RTX in the PB, with a transient peak early in the reconstitution. However, no correlation of plasma cell kinetics to time to relapse could be shown, which limits their usage for clinical monitoring [5,6].

Another candidate B-cell subset of relapsing autoimmunity might be CD27^+^IgD^+ ^non-switched MemB, which according to their surface marker expression are reported to correspond to splenic CD27^+^IgD^+^(IgM^+^) marginal zone B cells in rodents [25,26]. Cells of this developmental stage are able to undergo CD27-mediated co-stimulation but have not yet switched their Ig receptor isotype. They are not prone to the GC-related processes of antigen-dependent maturation but may undergo T-cell-independent maturation outside a lymphoid follicle. CD27^+^IgD^+ ^B cells are centrally involved in the processes of innate host defense, but on the other hand, they also represent several features that argue for a role in autoimmunity [27,28]. Their number was associated with RA relapse in the regenerating B-cell compartment in previous studies [5,6]. Like switched MemB, they may also develop to plasmablasts [20], which are able to secrete RF.

In this study, we questioned whether the advantage of individual RTX scheduling could be achieved by combining serological and cytological monitoring strategies. We confirm the previously reported importance of RF kinetics [1,7]. In addition, we found that, by using a B-cell monitoring strategy in CD45^+^CD19^+ ^B cells (Additional data file 1), sustained depletion of CD27^+^IgD^- ^class-switched MemB from the blood was associated with good clinical response to RTX treatment. We found also that the same B-cell subset, but not the CD27^+^IgD^+ ^non-switched MemB or the CD27^-^IgD^+ ^'naïve' B cells, were preferentially accumulated in actively inflamed joints.

## Materials and methods

### Patients

Thirty-five patients with RA according to the American College of Rheumatology classification criteria [29] were included in this prospective observational study. All patients were 'RTX-naïve'. They had active disease according to a 28-joint disease activity score (DAS28) of greater than 3.2, which would qualify them for repetitive RTX treatment [17]. All patients had failed to at least one disease-modifying antirheumatic drug (DMARD) and had shown inappropriate response to at least one tumour necrosis factor (TNF)-blocking agent. Disease activity was reflected by a median of 6 swollen (interquartile range [IQR] 3 to 10) and a median of 5 tender (IQR 2 to 9) of 28 evaluated joints. Median DAS28 was 5.0 (IQR 4.3 to 5.9), median erythrocyte sedimentation rate (ESR) was 33 mm (IQR 27 to 46), and C-reactive protein (CRP) serum concentration was 11 mg/L (IQR 3 to 24). Other patient characteristics, including the number of previously used DMARDs and anti-TNF agents, are summarized in Table 1. Assessors for clinical parameters were blinded to the time-matched laboratory results. All patients gave their written informed consent to participate. The study was approved by the Cantonal Ethics Committee of Bern (ref. no. 254/07).

**Table 1:** Baseline patient characteristics given in absolute number and percentage

Baseline patient characteristic	Absolute number	Percentage
Gender distribution (female)	30/35	85%
RF-positive	21/35	60%
CCP antibody-positive	21/35	60%
RF- and anti-CCP-positive	18/35	54%
Erosive disease	26/35	74%
Extra-articular manifestations 6/35 17% Age at disease onset, years	38^a^	
Age at inclusion, years	55^a^	
Disease duration at inclusion, months	108^a^	
Number of previous DMARDs	2.9^b^	
Number of previous anti-TNF agents	1.6^b^	
Concurrent MTX alone	22	63%
DMARD combination including MTX	2	6%
Leflunomide	4	11%
Prednisone dose, mg/day	10^a^	

^a^The median value is presented. ^b^The mean value is presented. CCP, cyclic citrullinated peptide; DMARD, disease-modifying antirheumatic drug; MTX, methotrexate; RF, rheumatoid factor; TNF, tumour necrosis factor.

### Treatment

B-cell depletion therapy was performed with two infusions of 1,000 mg RTX 14 days apart from each other, and both were co-administered with 100 mg prednisone in order to prevent allergic reactions. Low-dose methotrexate in stable weekly doses of between 10 and 25 mg and other conventional DMARDs were continued during the entire observation phase. Oral prednisone doses were maintained from baseline to month 3 with a median of 10 mg per day. Afterward, they could be adjusted to the clinical course, which resulted in median doses of 6 mg at month 3 and 5 mg at months 9 and 12. Corticosteroid doses were thus significantly lower (*P *< 0.05) in good responders than in moderate or non-responders at months 9 and 12.

### Response

Clinical improvement was assessed every 3 months by DAS28 and graded as European League Against Rheumatism (EULAR) good, moderate, or non-response [30]. EULAR good response, which could have been achieved at any visit during the 12-month observation period, was used for group definition in retrospective B-cell and antibody analyses.

### Sample preparation

Freshly isolated PB (9 mL) was anticoagulated with EDTA (ethylenediaminetetraacetic acid) and immediately used for flow cytometry. B-cell analyses were also performed in anticoagulated synovial fluids (n = 3) or in tissue homogenates from another three patients undergoing urgent joint replacement surgery of the knee (n = 1), synovectomy in treatment-resistant synovitis of the knee (n = 1), or wrist joint synovectomy (n = 1). The study protocol for invasive procedures was approved by the Cantonal Ethics Committee of Bern (254/07). All study participants gave their written informed consent to participate.

Synovial tissue was immediately prepared, as previously described [31], by injecting 1 mg/mL collagenase (Sigma-Aldrich, Munich, Germany) into the tissue samples, followed by incubation for 20 minutes at ambient temperature. Digests subsequently were minced and incubated for an additional 50 minutes at 37°C in collagenase 1%. The cell suspension was strained by 70-μm nylon filters (Falcon, now part of BD Biosciences, San Jose, CA, USA), washed twice in phosphate-buffered saline, and recovered in RPMI 1640 medium (Invitrogen, Karlsruhe, Germany) containing 10% fetal calf serum plus kanamycin at 37°C in 5% CO_2 _atmosphere overnight. Non-adherent synovial tissue cells were carefully obtained together with the supernatants, centrifuged, and thoroughly washed with phosphate-buffered saline before subsequent analyses.

### Flow cytometry

Fixation of leukocytes and lysis of erythrocytes for flow cytometry were done for quantitative analyses in TruCOUNT™ tubes (Becton Dickinson, Basel, Switzerland). Cells were stained with BD Multitest™ reagent for CD3/CD16 + CD56/CD45/CD19 markers as well as phycoerythrin-conjugated anti-CD27 (clone L128) and fluorescein isothiocyanate-conjugated anti-IgD (clone IA6-2). All antibodies were purchased from BD Biosciences. Data were acquired by flow cytometry using the BD FACSCalibur Flow Cytometry System and CellQuest software (BD Biosciences Immunocytometry Systems). Analyses were performed after gating on anti-CD45 stained lymphocytes. The number and frequency of CD19^+ ^PB B cells and subsets (percentage of CD19^+ ^B cells) were determined in a minimum of 20,000 events in the CD45 gate. Data collection was continued to 1 × 10^5 ^events in case there were fewer than 0.5% CD19^+ ^cells. The number and frequency of CD27^-^IgD^+ ^'naïve', CD27^+^IgD^+ ^non-switched MemB, and CD27^+^IgD^- ^switched MemB were determined according to their IgD and CD27 surface expression. All measurements were performed under the standard operating procedure guidelines of our accredited laboratory for flow cytometry.

### Serum antibodies

IgG, IgA, and IgM serum concentrations as well as serum RF were determined by nephelometry (Dade Behring Nephelometer; Dade Behring, Marburg, Germany). IgG antibodies against cyclic citrullinated peptides (CCPs) were also determined with commercially available methods (INOVA QUANTA Lite™ CCP3 ELISA; INOVA Diagnostics, Inc., San Diego, CA, USA).

### Statistics

Results are presented as median and 25% to 75% IQR for data in non-Gaussian distribution. The Wilcoxon rank sum test was used for comparison of two-tailed groups. The exact significance in the Mann-Whitney *U *test was used for comparison of two independent groups. Statistical analyses were performed with SPSS software version 15 (SPSS Inc., Chicago, IL, USA). Results with a *P *value of less than 0.05 were considered significant.

## Results

### Clinical response

Ten patients did not achieve a significant clinical improvement during the observation period. Six patients fulfilled the EULAR criteria for moderate clinical response, and another 19 for good clinical response. Best achieved clinical response, by definition, could have occurred at any time during the 12-month observation phase after RTX. Ten of our good-response patients fulfilled this EULAR definition for the first time after 3 months, another 7 patients after 6 months, and 2 patients only 9 months after RTX. Duration of good response was documented for a median of 3 months but in fact may have been substantially longer with regard to the 3-month visit intervals. The criterion of moderate response was documented continuously in these same 19 patients for a median of 9 months. Patients experiencing good response had significantly higher anti-CCP antibody titer (*P *= 0.046) at baseline but significantly lower CRP serum concentration (*P *= 0.010). They had significantly shorter disease duration (*P *= 0.008) and fewer DMARD treatment attempts (*P *= 0.010) but were comparable for ESR, RF, swollen and tender joint counts, corticosteroid dose, and DAS28 at baseline. Median CRP in the entire study population dropped significantly from 11 mg/L (IQR 3 to 24) at baseline to values below the detection limit of 3 mg/L, with an IQR of between less than 3 and 9 mg/L at month 3 (*P *= 0.019), less than 3 to 6 mg/L at month 6 (*P *< 0.001), and less than 3 to 7 mg/L at month 9 (*P *= 0.008). Good responders had significantly lower CRP serum concentrations than the other patients 6 months after RTX. ESR improved significantly from a median of 33 mm/hour (IQR 26 to 40) at baseline to a median of 11 mm/hour (IQR 7 to 25, *P *= 0.011) at month 3 and a median of 11 mm/hour (IQR 5 to 20, *P *= 0.006) at month 6. Good responders had significantly lower ESR than the other patients after 3, 6, and 12 months. After 12 months, median CRP concentrations as well as ESR were in the same range as baseline values.

### Composition of peripheral blood B cells at baseline

**Figure 1:**
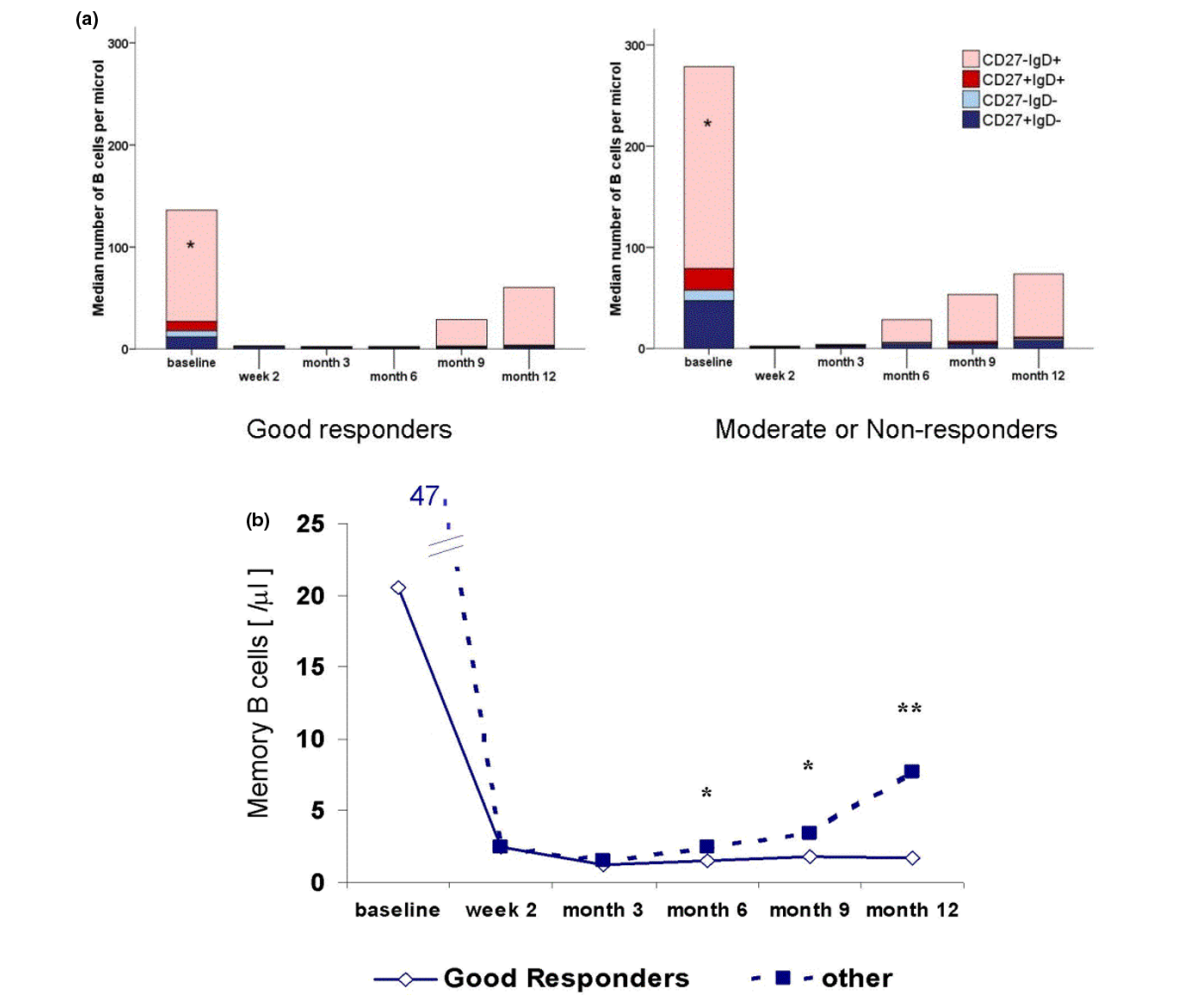
Peripheral blood B-cell repletion. **(a) **Median numbers of CD19^+ ^peripheral blood B cells in good responders (left) and non-responders or moderate responders (right) at different time points after B-cell depletion. Colours represent the median of different B-cell subsets: CD27^-^IgD^+ ^naïve B cells are in light red, CD27^+ ^IgD^+ ^non-switched memory B cells (MemB) are in red, CD27^-^IgD^- ^B cells are in light blue, and switched CD27^+^IgD^- ^MemB are in blue. **(b) **Line diagram of switched MemB in good responders (continuous line) and in non-responders or moderate responders (dotted line). Mann-Whitney test for comparison of good responders versus other patients: *P < 0.05; **P < 0.01.

Median total number of CD19^+ ^B cells (Figure 1) in the entire cohort was 169/μL (IQR 77 to 281); this parameter did not significantly differ between good responders and patients with moderate or no response in the later course. A median of 69% of B cells (IQR 55% to 79%) at baseline were 'naïve' B cells. Their absolute number was significantly higher (*P *= 0.034) in subsequent non-responders or moderate responders (median 199, IQR 137 to 297) than in good responders (median 109, IQR 56 to 142). In contrast, the frequencies of non-switched MemB (median 8%, IQR 4% to 13%), of switched MemB (median 16%, IQR 12% to 22%), and of CD27^-^IgD^- ^B cells (median 4%, IQR 3% to 7%), as well as their absolute numbers, were not significantly different between the two patient groups.

### Early response to depletion

The first RTX infusion reduced the number (Figure 1) and frequency of PB CD19^+ ^B cells to a median of 3/μL (IQR 2 to 7) and a median of 2% (IQR 1% to 4%) of CD45^+ ^cells, respectively. The median of all B-cell fractions decreased significantly in number (*P *< 0.001), but increasing frequency of switched MemB and of CD27^-^IgD^- ^B cells upon the first RTX infusion indicated relative resistance of these subsets. When a B-cell frequency of below 0.5% of lymphocytes [6] or the number of CD19^+ ^cells below 5/μL was used as an arbitrary surrogate of complete depletion, these values were reached in 61.3% and 48.6% of the cases, respectively, 14 days after the first infusion. Neither the achievement of one of these limits upon first infusion nor the absolute number or frequency of any defined B-cell subset in the early depletion phase was indicative for subsequent clinical response.

### Peripheral blood reconstitution

The median number of B cells increased continuously from months 3 to 12. This PB B-cell repletion was dominated by 'naïve' B cells. The time point of repopulation tended to be earlier in moderate or non-responders than in good responders; however, this difference was statistically not significant. In contrast, the absolute number of switched MemB at month 6 (*P *= 0.049), month 9 (*P *= 0.045), and month 12 (*P *= 0.003) was significantly higher in patients with no or moderate time-matched clinical response to RTX than in good responders (Figure 1). This result was the same when correlating switched MemB with best achieved response at any time of the treatment cycle. In contrast, the number and frequency of non-switched MemB, of CD27^-^IgD^-^, and of 'naïve' B cells were not correlated with the clinical response after RTX.

### Comparison of peripheral blood and synovial B cells

**Figure 2:**
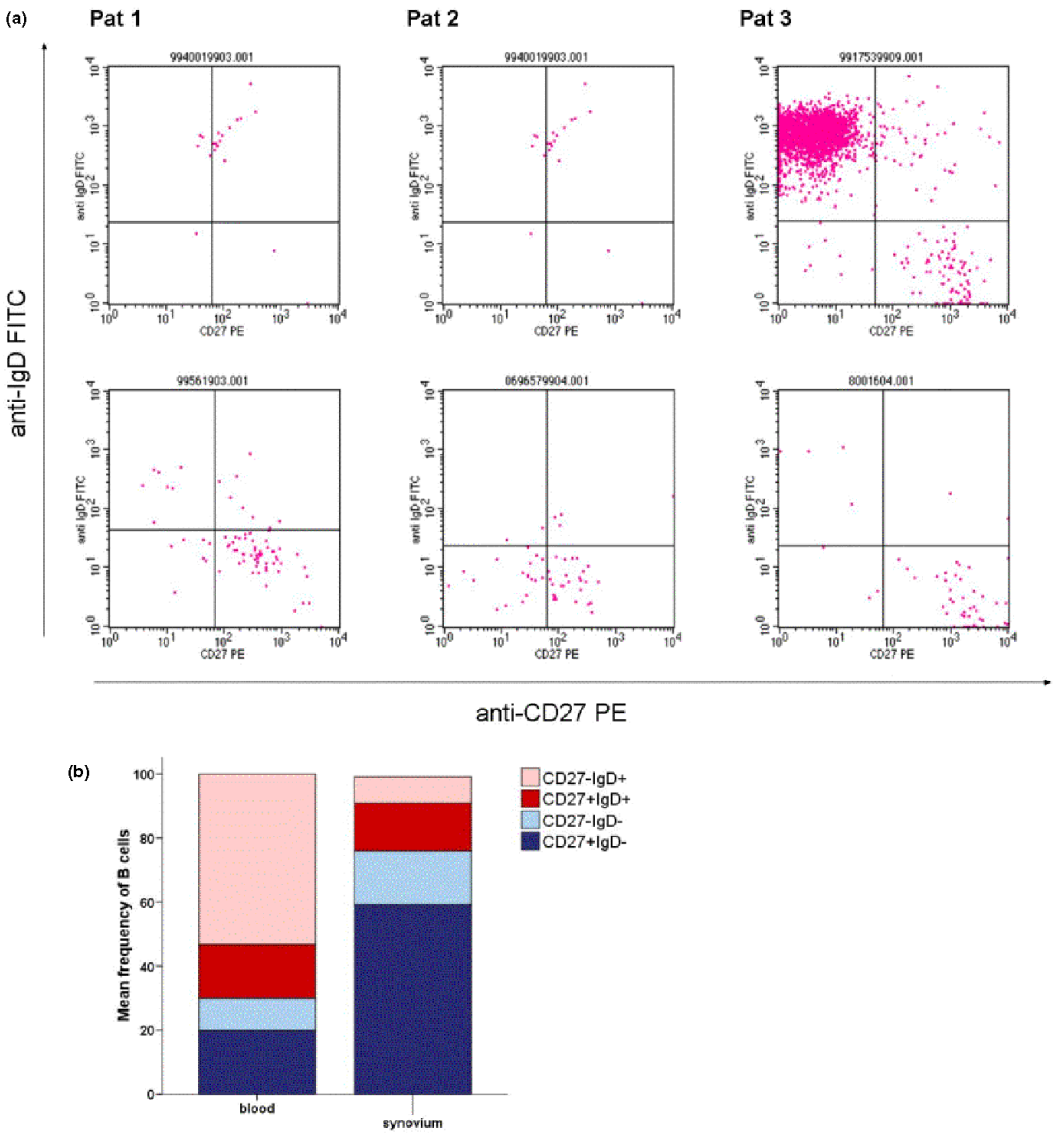
B-cell developmental stages in time-matched samples from the peripheral blood (PB) and synovium. **(a) **Original scatter plots of flow cytometric analyses on PB (top row) and synovial B cells (bottom row) in three different patients. CD27 staining is depicted on the horizontal axis, and IgD surface staining is depicted on the vertical axis. Quadrants are set according to the results in unstained cells for compensation of autofluorescence. Patient 1 (left column) had persistent arthritis 4 months after rituximab (RTX) and a PB B-cell count of 4/μL. Patient 2 (middle column) had arthritis relapse 10 months after RTX and a PB CD19^+ ^B-cell count of 27/μL. Patient 3 (right column) had arthritis relapse 9 months after RTX and a PB CD19^+ ^B-cell count of 71/μL. **(b) **Cumulative data on the frequency of B-cell subsets in six pairs of samples, including patients depicted in (a), indicate preferential accumulation of CD27^+^IgD^- ^switched memory B cells in the synovium (*P *= 0.028). FITC, fluorescein isothiocyanate; PE, phycoerythrin.

The B-cell composition in six simultaneously collected samples from the PB and synovium during B-cell repletion (Figure 2) gave further evidence for the importance of switched MemB: While 'naïve' B cells dominated quantitatively in PB during B-cell repletion, only the frequency of switched MemB was significantly higher in the synovium (*P *= 0.028) than in PB. A median of 50% (IQR 18% to 93%) of PB B cells, but only 8% (IQR 3% to 12%) of synovial B cells, were of 'naïve' phenotype, whereas 10% (IQR 10% to 41%) of PB B cells as compared with 67% (IQR 43% to 76%) of synovial B cells exhibited the CD27^+^IgD^- ^phenotype. (For histology, see Additional data file 2.)

### Rheumatoid factor and cyclic citrullinated peptide antibodies

**Figure 3:**
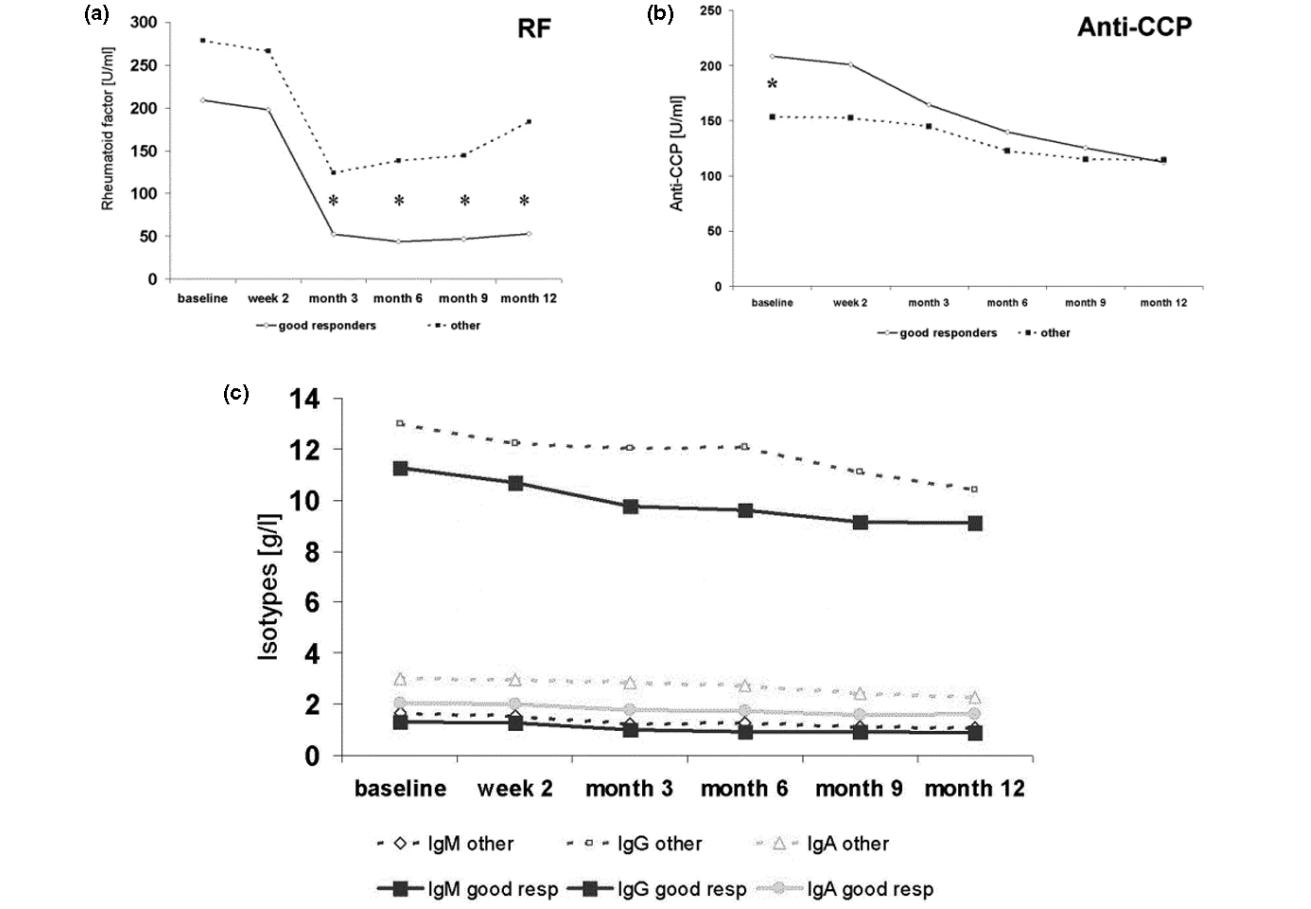
Antibody concentrations. Course of **(a) **rheumatoid factor (RF), **(b) **cyclic citrullinated peptide (CCP) antibodies, and **(c) **total immunoglobulin IgG, IgA, and IgM serum concentrations after therapeutic B-cell depletion in good responders (continuous line) and in non-responders or moderate responders (designated as other patients, dotted line). Asterisks indicate statistically significant differences (*P *< 0.05) between these two patient groups.

Therapeutic intervention with RTX led to significant decreases in RF and CCP antibody serum concentrations from months 3 to 12 (*P *< 0.05) (Figure 3). In patients who were RF^+ ^at baseline, the lowest RF concentration (median of 36% from baseline) was achieved after 6 months. Five of the 21 initially RF^+ ^patients became RF^- ^upon B-cell depletion, three of them lasting until the end of the observation whereas two others started to have positive RF tests again after 6 months. All patients with persistent or transient RF conversion were good clinical responders. A decrease of RF serum concentrations in comparison with baseline levels was significantly stronger in good responders than in the other patients. These results were similar for month 3 (*P *= 0.022), month 6 (*P *= 0.002), month 9 (*P *= 0.002), and after 12 months (*P *= 0.001). A significant reduction in median RF concentrations was long-lasting in good responders but was limited to a maximum of 6 months in moderate and non-responders. RF serum concentrations dropped more steeply than expected from the kinetics of slowly reduced IgG, IgA, and IgM total Ig concentrations.

In contrast to RF, CCP antibody concentrations decreased more continuously and in parallel to Ig levels. They reached their minimum of a median of 60% from baseline levels after 12 months. Good responders started with a tendency toward higher anti-CCP serum concentrations than the other patients but showed a significantly stronger reduction of CCP-directed autoantibodies after 9 months (*P *= 0.041) and 12 months (*P *= 0.027).

### Immunoglobulin isotypes

Total IgG serum concentrations started to be significantly reduced (*P *< 0.05) already after the first infusion, whereas IgA and IgM serum concentrations were more stable. These isotypes came down somewhat later, with significantly reduced levels from months 3 to 12 (*P *< 0.001) when compared with baseline. Three different patients marginally underwent the lower limit for normal IgG (7 g/L) or IgA (0.7 g/L) serum concentrations during the observation phase. Two other patients developed IgM concentrations below the lower normal limit of 0.4 g/L. None of the patients with a drop of any Ig isotype below the lower normal limit developed clinical symptoms of immunodeficiency, but seven of these patients experienced good clinical response and one patient experienced moderate clinical response.

After 12 months, the IgG and IgA serum concentrations decreased to medians of 86% and 79% and IgM serum concentrations decreased to values between 75% and 68% from baseline. Median IgG, IgA, and IgM serum concentrations were somewhat lower in good responders at any follow-up visit, but at no time point were the relative decreases of these concentrations from baseline significantly different between good responders and the other patients.

## Discussion

Convincing clinical success of therapeutic depletion brought the B cells back into the research focus of RA pathogenesis. The present data indicate that concentrations of one of their products, serum RF, as well as the repletion kinetics of switched MemB in the blood and their migration into joints are linked to inflammatory activity. In the following sections, we will discuss the impact of these possibly linked findings on our understanding of RA pathogenesis and their potential for scheduling re-treatment.

Identical B-cell clones in different joints and the blood of RA patients reflect the systemic autoimmune character of RA [32]. While PB B cells become rapidly depleted after RTX, improvement in RA symptoms is delayed. This disconnection in time might suggest similar B-cell persistence mechanisms in the RA synovium, as recently described for splenic marginal zone and GC B cells in Peyer's patches in a rodent anti-human CD20 depletion model [33]. Given these data, it was important to prove in synovial biopsy studies that CD20^+ ^B cells could be depleted, though to various degrees and rapidities, in the inflamed synovial microenvironment [21-24]. Reports on the size and number of lymphoid aggregates were also somewhat contradictory [21,23]. Clinical improvement upon RTX was correlated with a decrease in the number of synovial B cells in one biopsy study [22] and with reduced plasma cell numbers in another biopsy study [23]. In summary and according to the recently formulated 'roadblock hypothesis' [34], histology data after RTX draw the picture of an ongoing process of renewing B cells in the synovium which can be interrupted by therapeutic intervention.

As a consequence of B-cell depletion, the synovial Ig production (including RF and CCP specificities) decreased in quantity. The effect on RF and anti-CCP idiotypes appeared less or even absent in aggregated lymphocellular infiltrates [24,35], which was the histological subtype with the highest autoantibody production [35]. So far, these data appear to be in accordance with high IgM anti-CCP serum antibodies and with RF titer, which together with high-grade synovial CD20^-^CD79a^+ ^B-cell infiltrates were negative predictors for RTX response in another longitudinal analysis [22]. Negative data in cross-sectional analyses of the same serological and histological parameters draw a close connection of these prognostic items into question [36]. Good responders among our patients were characterised by significantly higher numbers of 'naïve' B cells in PB and also by higher CCP serum antibody and lower CRP concentrations at baseline. Given that we searched for statistical significance of difference for many comparisons, a statistical error of multiple testing has to be considered. In addition, as the same items were previously examined in similar settings without providing support for our observations, evidence appears to be too weak for recommending the determination of 'naïve' B cells as a predictor of response to RTX.

We confirmed [1] in this study that serum RF decreases faster and to lower relative levels than expected from the corresponding IgM isotype kinetics. Thus, RF-producing B cells were more sensitive to RTX than B cells of other specificities. A more lasting reduction of RF titer in good responders when compared with less favourably responding patients furthermore indicates that the depletion of RF-expressing cells is more profound and might have direct therapeutic impact. In contrast, the kinetics of anti-CCP antibodies followed, with large inter-individual variation and despite their well-established diagnostic and prognostic roles in RA [37-39], just the corresponding isotype concentrations. This finding is confirmatory of previous studies [7,23]. RF-expressing B cells may act pleiotropic, but they provide a function with unique consequences for the involvement of multiple antigens as seen in RA [10]: Their receptors can complex Igs of different specificity, together with any bound autoantigen or foreign antigen. Processing of these complexes and antigen presentation to their T-cell counterparts may lead to affinity-matured, class-switched MemB for a variety of specificities [12]. It appears likely, following this line of argumentation, that the profound reductions of GC-derived memory and (in parallel) of RF-producing B cells 6, 9, and 12 months after RTX were therapeutically relevant and linked processes.

Murine models for studies on self-reactive B cells localised the failure of tolerance mechanisms in the bone marrow [40]. Although the secondary lymphoid organs examined in an animal model may differ importantly from lymphoid neogenesis observed in RA synovitis [9,33], GC-forming B cells appear essential for mutual B-cell and T-cell activation and for pro-inflammatory cytokine response in human synovitis [41]. T-cell-dependent affinity maturation of RF-producing B cells is anatomically linked to the GCs of secondary immune organs [42-44], but non-switched MemB, another source of RF, do not require lymphoid aggregates [9,19]. It appears notable, in this context and with regard to our data, to recall the higher specificity of IgA RF and its impact on the RA disease course in comparison with non-switched RF [45,46]. As close antigen and major histocompatibility complex (MHC)-restricted B cell-T cell interaction is responsible for shaping of the receptor repertoire by somatic hypermutation of the B-cell receptor, this process is obviously of critical importance for the induction of autoimmune B cells. It was shown in a recent publication that somatic hypermutation in RA is predominantly operative in CD19^+^IgD^- ^class-switched B cells and that the frequency of such mutated B cells is substantially modified by RTX treatment [47]. Thus, therapeutic interruption even of abortive GCs appears promising.

PB B cells started to repopulate in all of our patients during the first 12 months after RTX. It is currently unknown whether the reoccurrence of somatically mutated plasmablasts in the PB in the early repletion phase is a recirculation phenomenon from their survival niches or the result of rapid *de novo *differentiation. In accordance with the literature [4-6], it was neither time of reoccurrence nor the numbers of total B cells or quantitatively dominant 'naïve' B cells in PB in our study that were correlated with best achieved or time-matched clinical response. The same was true for CD27^+^IgD^+ ^and CD27^-^IgD^- ^B cells, which thereby appeared to be irrelevant for clinical monitoring. In contrast, robust data on the course of switched MemB in relation to disease indicate that their monitoring might find a place in clinical application as an alternative to or might add to quantitative RF analyses [12]. These data on a level of statistical significance are essentially in agreement with reports about a trend toward shorter time between B-cell repopulation and clinical relapse in patients reconstituting their PB B-cell compartment with a higher proportion of switched MemB [4,6]. Finally, accumulation of switched MemB in the synovium, the end organ of immune-mediated processes, underscores the relevance of this B-cell subset in RA. Taken together, these data indicate that synovial lymphoid structure formation depends on trafficking of circulating rather than on locally expanding B cells, thereby allowing B-cell monitoring after RTX not only in the synovium, but also in the blood.

When median DAS28 values increased continuously from 2.1 to 2.8 in good responders and from 3.3 to 4.5 in the other patients, the approximate cutoff values of switched MemB in the blood between the two groups of our study were two cells per microlitre after 6 months, four cells per microlitre after 9 months, and six cells per microlitre 12 months after RTX. In analyses of all available data irrespective of the time after RTX, the upper cutoff value for patients in DAS28 remission (< 2.6) was also two CD27^+^IgD^- ^B cells per microlitre. Persistence of the CD27^+^IgD^- ^B-cell subset above this threshold indicated resistance to RTX, while exceeding the threshold after good response was associated with disease relapse. We estimate that it is unlikely that lower doses of concomitant steroids in good responders were responsible for this observation. Therefore, it would appear to be worthwhile to prospectively test the clinical outcome of repetitive B-cell depletion on the basis of the proposed monitoring procedure in comparison with other strategies.

## Conclusions

Our data indicate that B-cell maturation in the PB after RTX does not just display lymphatic ontogeny [4,15] but provides clinically relevant data when focussing on switched MemB. Clarifying whether the disease-related cell phenomena are sufficiently antecedent to a clinical flare to be useful in clinical practice requires ongoing research in a prospective setting.

## Abbreviations

CCP: cyclic citrullinated peptide; CRP: C-reactive protein; DAS28: 28-joint disease activity score; DMARD: disease-modifying antirheumatic drug; ESR: erythrocyte sedimentation rate; EULAR: European League Against Rheumatism, GC: germinal centre; Ig: immunoglobulin; IQR: interquartile range; MemB: memory B cell; PB: peripheral blood; RA: rheumatoid arthritis; RF: rheumatoid factor; RTX: rituximab; TNF: tumour necrosis factor.

## Competing interests

Roche Pharmaceuticals Switzerland (Basel, Switzerland) supported this study with an unrestricted research grant of 30,000 Swiss Francs. Roche did not have any influence on the collection, evaluation, or interpretation of the data or on the preparation of this manuscript.

## Authors' contributions

BM supervised data collection, performed statistical analyses, and wrote the manuscript and takes the collective responsibility for the integrity of the data and conclusions. CAD supervised and analysed the cellular and serologic tests. SE and EV provided the synovial samples. IV performed immunohistochemistry. MF contributed to data analysis and manuscript preparation. DA, H-RZ, and PMV contributed to patient recruitment and clinical data collection. All authors were actively involved in the drafting of the manuscript and in critical revision. All authors read and approved the final manuscript.

## Supplementary Material

Additional data file 1 Schematic overview of B cell developmental stages (names in boxes) and corresponding surface markers (in boxes with punctured lines) that were used in this study. Immunoglobulin class-switch of B cells is functionally linked with antigen dependent, MHC restricted affinity maturation, and anatomically related to germinal centre (GC) formation. CD27+IgD- class-switched B cells can either be post-germinal centre memory B cells, or plasmablasts, which are directed to further plasma cell development. We show in this study that the kinetics of class-switched B cells is associated with the course of RA disease activity. MHC-II: class 2 major histocompatibility complex, TCR: T cell receptor, CD27 and CD70: TNF-α family members and co-stimulatory molecules on B cells and T cells. Click here for file

Additional data file 2 Histological preparations from RA synovitis in the early B cell reconstitution phase after RTX. Haematoxylin-eosin staining and immunohistochemistry for synovial B cells during the early peripheral blood B cell repletion phase. Flow cytometric analyses for IgD and CD27 expression from the same sample are depicted as patient 3 in Figure 2A. The infiltrating CD20+ B cells form a few small lymphoid aggregates with large CD79a+ CD20- plasma cells (arrows). This exemplary staining was performed in a synovial sample from a flaring knee joint using CD20 (clone L26) and CD79a antibodies (clone JCB117) from Dako, Glostrup, Denmark. Immunohistochemistry slides have been obtained using a three-step streptavidin-biotin technique, and new-fuchsin as chromogen. Original magnification × 200. Click here for file

## References

[B1] Edwards JC, Szczepanski L, Szechinski J, Filipowicz-Sosnowska A, Emery P, Close DR, Stevens RM, Shaw T (2004). Efficacy of B-cell-targeted therapy with rituximab in patients with rheumatoid arthritis. N Engl J Med.

[B2] Emery P, Fleischmann R, Filipowicz-Sosnowska A, Schechtman J, Szczepanski L, Kavanaugh A, Racewicz AJ, van Vollenhoven RF, Li NF, Agarwal S, Hessey EW, Shaw TM, DANCER Study Group (2006). The efficacy and safety of rituximab in patients with active rheumatoid arthritis despite methotrexate treatment: results of a phase IIB randomized, double-blind, placebo-controlled, dose-ranging trial. Arthritis Rheum.

[B3] Cohen SB, Emery P, Greenwald MW, Dougados M, Furie RA, Genovese MC, Keystone EC, Loveless JE, Burmester GR, Cravets MW, Shaw T, Totoritis MC, REFLEX Trial Group (2006). Rituximab for rheumatoid arthritis refractory to anti-tumor necrosis factor therapy: Results of a multicenter, randomized, double-blind, placebo-controlled, phase III trial evaluating primary efficacy and safety at twenty-four weeks. Arthritis Rheum.

[B4] Leandro MJ, Cambridge G, Ehrenstein MR, Edwards JC (2006). Reconstitution of peripheral blood B cells after depletion with rituximab in patients with rheumatoid arthritis. Arthritis Rheum.

[B5] Roll P, Palanichamy A, Kneitz C, Dorner T, Tony HP (2006). Regeneration of B cell subsets after transient B cell depletion using anti-CD20 antibodies in rheumatoid arthritis. Arthritis Rheum.

[B6] Roll P, Dorner T, Tony HP (2008). Anti-CD20 therapy in patients with rheumatoid arthritis: Predictors of response and B cell subset regeneration after repeated treatment. Arthritis Rheum.

[B7] Cambridge G, Leandro MJ, Edwards JC, Ehrenstein MR, Salden M, Bodman-Smith M, Webster AD (2003). Serologic changes following B lymphocyte depletion therapy for rheumatoid arthritis. Arthritis Rheum.

[B8] Taylor RP, Lindorfer MA (2007). Drug insight: the mechanism of action of rituximab in autoimmune disease – the immune complex decoy hypothesis. Nat Clin Pract Rheumatol.

[B9] Cantaert T, Kolln J, Timmer T, Kraan TC van der Pouw, Vandooren B, Thurlings RM, Canete JD, Catrina AI, Out T, Verweij CL, Zhang Y, Tak PP, Baeten D (2008). B lymphocyte autoimmunity in rheumatoid synovitis is independent of ectopic lymphoid neogenesis. J Immunol.

[B10] Corrigall VM, Panayi GS (2002). Autoantigens and immune pathways in rheumatoid arthritis. Crit Rev Immunol.

[B11] Keystone E, Fleischmann R, Emery P, Furst DE, van Vollenhoven R, Bathon J, Dougados M, Baldassare A, Ferraccioli G, Chubick A, Udell J, Cravets MW, Agarwal S, Cooper S, Magrini F (2007). Safety and efficacy of additional courses of rituximab in patients with active rheumatoid arthritis: an open-label extension analysis. Arthritis Rheum.

[B12] Edwards JC, Cambridge G (2006). B-cell targeting in rheumatoid arthritis and other autoimmune diseases. Nat Rev Immunol.

[B13] Kolk LE van der, Baars JW, Prins MH, van Oers MH (2002). Rituximab treatment results in impaired secondary humoral immune responsiveness. Blood.

[B14] Popa C, Leandro MJ, Cambridge G, Edwards JC (2007). Repeated B lymphocyte depletion with rituximab in rheumatoid arthritis over 7 yrs. Rheumatology (Oxford).

[B15] Anolik JH, Friedberg JW, Zheng B, Barnard J, Owen T, Cushing E, Kelly J, Milner EC, Fisher RI, Sanz I (2007). B cell reconstitution after rituximab treatment of lymphoma recapitulates B cell ontogeny. Clin Immunol.

[B16] Leandro MJ, Cooper N, Cambridge G, Ehrenstein MR, Edwards JC (2007). Bone marrow B-lineage cells in patients with rheumatoid arthritis following rituximab therapy. Rheumatology (Oxford).

[B17] Smolen JS, Keystone EC, Emery P, Breedveld FC, Betteridge N, Burmester GR, Dougados M, Ferraccioli G, Jaeger U, Klareskog L, Kvien TK, Martin-Mola E, Pavelka K, Working Group on the Rituximab Consensus Statement (2007). Consensus statement on the use of rituximab in patients with rheumatoid arthritis. Ann Rheum Dis.

[B18] Radbruch A, Muehlinghaus G, Luger EO, Inamine A, Smith KG, Dorner T, Hiepe F (2006). Competence and competition: the challenge of becoming a long-lived plasma cell. Nat Rev Immunol.

[B19] William J, Euler C, Shlomchik MJ (2005). Short-lived plasmablasts dominate the early spontaneous rheumatoid factor response: differentiation pathways, hypermutating cell types, and affinity maturation outside the germinal center. J Immunol.

[B20] Sze DM, Toellner KM, Garcia de Vinuesa C, Taylor DR, MacLennan IC (2000). Intrinsic constraint on plasmablast growth and extrinsic limits of plasma cell survival. J Exp Med.

[B21] Vos K, Thurlings RM, Wijbrandts CA, van Schaardenburg D, Gerlag DM, Tak PP (2007). Early effects of rituximab on the synovial cell infiltrate in patients with rheumatoid arthritis. Arthritis Rheum.

[B22] Teng YK, Levarht EW, Hashemi M, Bajema IM, Toes RE, Huizinga TW, van Laar JM (2007). Immunohistochemical analysis as a means to predict responsiveness to rituximab treatment. Arthritis Rheum.

[B23] Thurlings RM, Vos K, Wijbrandts CA, Zwinderman AH, Gerlag DM, Tak PP (2008). Synovial tissue response to rituximab: mechanism of action and identification of biomarkers of response. Ann Rheum Dis.

[B24] Kavanaugh A, Rosengren S, Lee SJ, Hammaker D, Firestein GS, Kalunian K, Wei N, Boyle DL (2008). Assessment of rituximab's immunomodulatory synovial effects (ARISE trial). 1: clinical and synovial biomarker results. Ann Rheum Dis.

[B25] Pillai S, Cariappa A, Moran ST (2005). Marginal zone B cells. Annu Rev Immunol.

[B26] Weller S, Braun MC, Tan BK, Rosenwald A, Cordier C, Conley ME, Plebani A, Kumararatne DS, Bonnet D, Tournilhac O, Tchernia G, Steiniger B, Staudt LM, Casanova JL, Reynaud CA, Weill JC (2004). Human blood IgM 'memory' B cells are circulating splenic marginal zone B cells harboring a prediversified immunoglobulin repertoire. Blood.

[B27] Mandik-Nayak L, Racz J, Sleckman BP, Allen PM (2006). Autoreactive marginal zone B cells are spontaneously activated but lymph node B cells require T cell help. J Exp Med.

[B28] Odendahl M, Jacobi A, Hansen A, Feist E, Hiepe F, Burmester GR, Lipsky PE, Radbruch A, Dorner T (2000). Disturbed peripheral B lymphocyte homeostasis in systemic lupus erythematosus. J Immunol.

[B29] Arnett FC, Edworthy SM, Bloch DA, McShane DJ, Fries JF, Cooper NS, Healey LA, Kaplan SR, Liang MH, Luthra HS, Medsger TA, Mitchell DM, Neustadt DH, Pinals RS, Schaller JG, Sharp JT, Wilder RL, Hunder GG (1988). The American Rheumatism Association 1987 revised criteria for the classification of rheumatoid arthritis. Arthritis Rheum.

[B30] Fransen J, van Riel PL (2005). The Disease Activity Score and the EULAR response criteria. Clin Exp Rheumatol.

[B31] Behrens F, Himsel A, Rehart S, Stanczyk J, Beutel B, Zimmermann SY, Koehl U, Moller B, Gay S, Kaltwasser JP, Pfeilschifter JM, Radeke HH (2007). Imbalance in distribution of functional autologous regulatory T cells in rheumatoid arthritis. Ann Rheum Dis.

[B32] Voswinkel J, Weisgerber K, Pfreundschuh M, Gause A (1999). The B lymphocyte in rheumatoid arthritis: recirculation of B lymphocytes between different joints and blood. Autoimmunity.

[B33] Gong Q, Ou Q, Ye S, Lee WP, Cornelius J, Diehl L, Lin WY, Hu Z, Lu Y, Chen Y, Wu Y, Meng YG, Gribling P, Lin Z, Nguyen K, Tran T, Zhang Y, Rosen H, Martin F, Chan AC (2005). Importance of cellular microenvironment and circulatory dynamics in B cell immunotherapy. J Immunol.

[B34] Silverman GJ, Boyle DL (2008). Understanding the mechanistic basis in rheumatoid arthritis for clinical response to anti-CD20 therapy: the B-cell roadblock hypothesis. Immunol Rev.

[B35] Rosengren S, Wei N, Kalunian KC, Zvaifler NJ, Kavanaugh A, Boyle DL (2008). Elevated autoantibody content in rheumatoid arthritis synovia with lymphoid aggregates and the effect of rituximab. Arthritis Res Ther.

[B36] Thurlings RM, Wijbrandts CA, Mebius RE, Cantaert T, Dinant HJ, Pouw-Kraan TC van der, Verweij CL, Baeten D, Tak PP (2008). Synovial lymphoid neogenesis does not define a specific clinical rheumatoid arthritis phenotype. Arthritis Rheum.

[B37] Goldbach-Mansky R, Lee J, McCoy A, Hoxworth J, Yarboro C, Smolen JS, Steiner G, Rosen A, Zhang C, Menard HA, Zhou ZJ, Palosuo T, Van Venrooij WJ, Wilder RL, Klippel JH, Schumacher HR, El-Gabalawy HS (2000). Rheumatoid arthritis associated autoantibodies in patients with synovitis of recent onset. Arthritis Res.

[B38] Courvoisier N, Dougados M, Cantagrel A, Goupille P, Meyer O, Sibilia J, Daures JP, Combe B (2008). Prognostic factors of 10-year radiographic outcome in early rheumatoid arthritis: a prospective study. Arthritis Res Ther.

[B39] van Oosterhout M, Bajema I, Levarht EW, Toes RE, Huizinga TW, van Laar JM (2008). Differences in synovial tissue infiltrates between anti-cyclic citrullinated peptide-positive rheumatoid arthritis and anti-cyclic citrullinated peptide-negative rheumatoid arthritis. Arthritis Rheum.

[B40] Halverson R, Torres RM, Pelanda R (2004). Receptor editing is the main mechanism of B cell tolerance toward membrane antigens. Nat Immunol.

[B41] Takemura S, Klimiuk PA, Braun A, Goronzy JJ, Weyand CM (2001). T cell activation in rheumatoid synovium is B cell dependent. J Immunol.

[B42] Reparon-Schuijt CC, van Esch WJ, van Kooten C, Levarht EW, Breedveld FC, Verweij CL (1998). Functional analysis of rheumatoid factor-producing B cells from the synovial fluid of rheumatoid arthritis patients. Arthritis Rheum.

[B43] Duquerroy S, Stura EA, Bressanelli S, Fabiane SM, Vaney MC, Beale D, Hamon M, Casali P, Rey FA, Sutton BJ, Taussig MJ (2007). Crystal structure of a human autoimmune complex between IgM rheumatoid factor RF61 and IgG1 Fc reveals a novel epitope and evidence for affinity maturation. J Mol Biol.

[B44] Randen I, Brown D, Thompson KM, Hughes-Jones N, Pascual V, Victor K, Capra JD, Forre O, Natvig JB (1992). Clonally related IgM rheumatoid factors undergo affinity maturation in the rheumatoid synovial tissue. J Immunol.

[B45] Jonsson T, Valdimarsson H (1998). What about IgA rheumatoid factor in rheumatoid arthritis?. Ann Rheum Dis.

[B46] Bobbio-Pallavicini F, Caporali R, Alpini C, Avalle S, Epis OM, Klersy C, Montecucco C (2007). High IgA rheumatoid factor levels are associated with poor clinical response to tumour necrosis factor alpha inhibitors in rheumatoid arthritis. Ann Rheum Dis.

[B47] Palanichamy A, Roll P, Theiss R, Dorner T, Tony HP (2008). Modulation of molecular imprints in the antigen-experienced B cell repertoire by rituximab. Arthritis Rheum.

