# Supplementary figures and images for: Class-switched B cells display response to therapeutic B-cell depletion in rheumatoid arthritis
Source: Arthritis Res Ther. 2009 May 6;11(3):R62. doi: 10.1186/ar2686 (PMC2714106; doi:10.1186/ar2686)

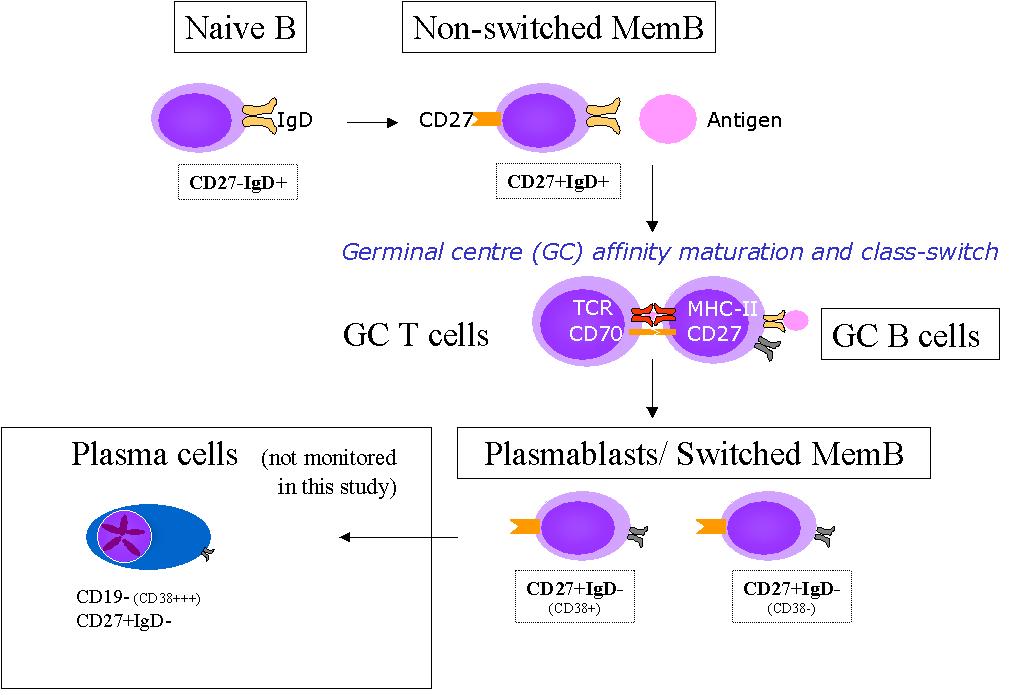

Supplement: Additional data file 1 — Schematic overview of B cell developmental stages (names in boxes) and corresponding surface markers (in boxes with punctured lines) that were used in this study. Immunoglobulin class-switch of B cells is functionally linked with antigen dependent, MHC restricted affinity maturation, and anatomically related to germinal centre (GC) formation. CD27+IgD- class-switched B cells can either be post-germinal centre memory B cells, or plasmablasts, which are directed to further plasma cell development. We show in this study that the kinetics of class-switched B cells is associated with the course of RA disease activity. MHC-II: class 2 major histocompatibility complex, TCR: T cell receptor, CD27 and CD70: TNF-α family members and co-stimulatory molecules on B cells and T cells. [file ar2686-S1.jpeg]

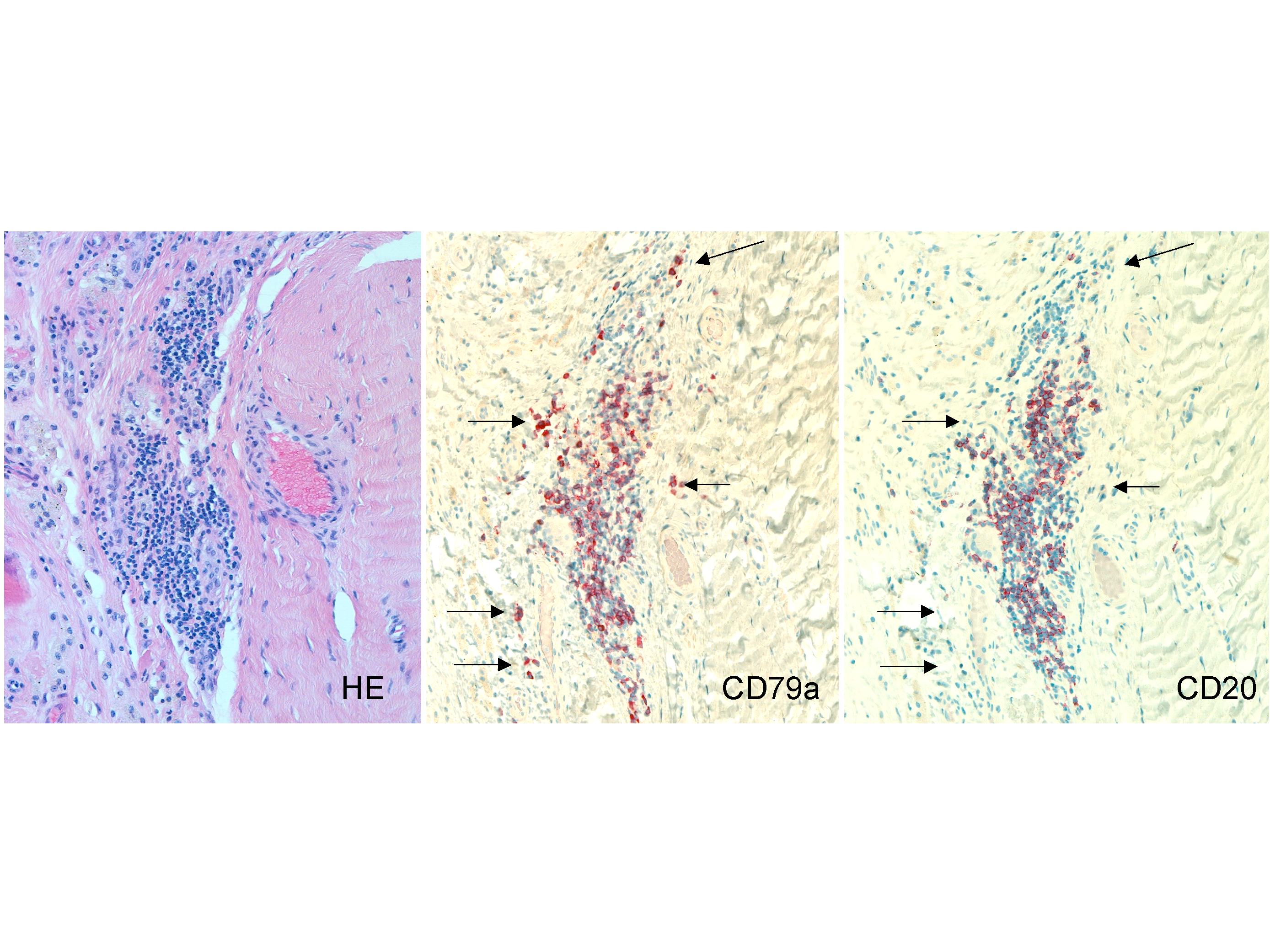

Supplement: Additional data file 2 — Histological preparations from RA synovitis in the early B cell reconstitution phase after RTX. Haematoxylin-eosin staining and immunohistochemistry for synovial B cells during the early peripheral blood B cell repletion phase. Flow cytometric analyses for IgD and CD27 expression from the same sample are depicted as patient 3 in Figure 2A. The infiltrating CD20+ B cells form a few small lymphoid aggregates with large CD79a+ CD20- plasma cells (arrows). This exemplary staining was performed in a synovial sample from a flaring knee joint using CD20 (clone L26) and CD79a antibodies (clone JCB117) from Dako, Glostrup, Denmark. Immunohistochemistry slides have been obtained using a three-step streptavidin-biotin technique, and new-fuchsin as chromogen. Original magnification × 200. [file ar2686-S2.bmp]
